# Initial Testing of an Approximated, Fast Calculation Procedure for Personalized Dosimetry in Radionuclide Therapy Based on Planar Whole-Body Scan and Monte-Carlo Specific Dose Rates from the *OpenDose* Project

**DOI:** 10.3390/life12091303

**Published:** 2022-08-25

**Authors:** Davide Bianco, Carmela Nappi, Leandra Piscopo, Fabio Volpe, Mariarosaria Manganelli, Federica Volpicelli, Filomena Loffredo, Pasquale Totaro, Maria Quarto, Alberto Cuocolo, Michele Klain

**Affiliations:** 1 Department of Advanced Biomedical Sciences, University “Federico II”, 80131 Naples, Italy; 2 Italian Aerospace Research Centre (CIRA), 81043 Capua, Italy

**Keywords:** radionuclide therapy, individualized dosimetry, planar WBS

## Abstract

Individualized dosimetry in nuclear medicine is currently at least advisable in order to obtain the best risk–benefit balance in terms of the maximal dose to lesions and under-threshold doses to radiosensitive organs. This article aims to propose a procedure for fast dosimetric calculations based on planar whole-body scintigraphy (WBS) images and developed to be employed in everyday clinical practice. Methods: For simplicity and legacy reasons, the method is based on planar imaging dosimetry, complemented with some assumptions on the radiopharmaceutical kinetics empirically derived from single-photon emission tomography/computed tomography (SPECT/CT) image analysis. The idea is to exploit a rough estimate of the time-integrated activity as has been suggested for SPECT/CT dosimetry but using planar images. The resulting further reduction in dose estimation accuracy is moderated by the use of a high-precision Monte-Carlo S-factor, such as those available within the OpenDose project. Results: We moved the problem of individualized dosimetry to a transformed space where comparing doses was imparted to the ICRP Average Male/Female computational phantom, resulting from an activity distribution related to patient’s pharmaceutical uptake. This is a fast method for the personalized dosimetric evaluation of radionuclide therapy, bearing in mind that the resulting doses are meaningful in comparison with thresholds calculated in the same framework. Conclusion: The simplified scheme proposed here can help the community, or even the single physician, establish a quantitative guide-for-the-eye approach to individualized dosimetry.

## 1. Introduction

Therapeutic approaches in nuclear medicine have always been faced with the need for activity optimization, consisting of maximizing the radiation dose to lesions while limiting that to healthy radiosensitive tissues, in particular the bone marrow (BM). Since the early introduction of radioactive iodine (RAI) therapy, consisting of the systemic administration of 131I-sodium or potassium iodide in patients with differentiated thyroid cancer (DTC) for remnant ablation after total thyroidectomy and metastases treatment [1], the evaluation of the dose imparted to normal tissues and tumors has been the subject of several studies [2,3,4]. The experimental evaluation of the activity retained within the blood for estimating the dose delivered to the BM was proposed in the seminal work of Benua [5,6,7], whose protocol has, for a long time, represented the gold standard in BM therapeutic dose evaluations.

Subsequently, the wide spreading of computer technologies in clinical practice allowed the collection and storage of whole-body scan (WBS) and single-photon emission tomography (SPECT) data in a digital format, leading to the development of a number of approaches to the dosimetry of nontargeted organs and tumors, based on the Committee for Medical Internal Radiation Dose (MIRD) formalism [8,9,10,11,12,13,14,15]. In the MIRD formalism [16], the total mean absorbed dose to the target region, $D\left(r_{T}\right)$, is computed by summing the separate contributions from each source region,$\;r_{S}$, including $r_{T}$ itself:

(1) $$D\left(r_{T}\right)=\underset{r_{S}}{\sum }\tilde{A}\left(r_{S}\right)S\left(r_{T}\leftarrow r_{S}\right)\;$$

where $\tilde{A}\left(r_{S}\right)$ indicates the cumulated time activity in $r_{S}$ and the S factor represents the absorbed dose rate to the target, $r_{T}$, per unit of activity retained in $r_{S}$.

In the last decade, the adoption of new radiopharmaceuticals, such as those employed in peptide receptor radionuclide therapy (PRRT), and the introduction of hybrid SPECT/CT scanners allowing voxel-based evaluations have increased the attention on dosimetry in nuclear medicine [17,18,19,20]. Methods using SPECT/CT data alone [21] or in combination with planar WBS images [22,23,24] have been developed to enhance the accuracy and personalization of dose calculation. In particular, the method prosed by Swenson, Hagmarker, and co-workers [23,24] employs an automatic segmentation algorithm that was originally developed in a different context [25].

Despite software and hardware implementation and the large amount of research carried out, detailed dosimetric evaluation in nuclear medicine therapies is still highly demanding in terms of single-exam acquisition time, longer hospitalization, and the need for serial tests [26,27]. Recently, it was suggested that repeated activity measurements might be avoided using a single SPECT/CT scan and pharmacokinetic parameters derived from experience [28]. Here, we present an approximated calculation scheme derived from the single-time-point approach described in [28], coupled with the two-compartments method employed in [25], where a segmentation of the whole-body planar image into high- and low-uptake areas is suggested for dosimetric purposes. This means that the required pharmacokinetic parameters, condensed within a scale factor for the time–activity curve, are derived in our method from a single WBS, substituting the single SPECT/CT proposed by Hänscheid [28]. The potential loss of accuracy is complemented using the detailed specific dose rate factors, S, available within the OpenDose project [29] for the average male and female phantoms.

The proposed approach, applied to the calculation of the dose imparted to the bone marrow, is based on a number of assumptions that will be discussed in detail in the rest of the document. They are summarized here for the sake of clarity.

The time–activity curve for the two compartments has a fixed shape, but it is scaled through a factor depending on the single time point measurements; this procedure is extremely fast and saves machine and personnel time but introduces an error in the cumulated activity reconstruction.

Scale factors are derived with a self-calibration approach, allowing a simplified quantification of the activity content, although anatomical regions with densities considerably differing from the mean body density, such as the lungs, are more difficult to treat. 

When there is not a specific uptake for the radiopharmaceutical in the blood and/or red marrow cells, as is the case with ^177^Lu-DOTA-TATE and ^131^I, a fixed ratio between activity concentrations in the bone marrow and the low-uptake compartment is postulated; this simplification avoids the use of a SPECT scan but reduces the patient specificity of the dose calculation. 

The method has been preliminarily validated against literature data from PRRT. As a second end point, the comparison of BM dose calculated with the proposed method and with the Benua approach has been carried out in five patients with DTC undergoing RAI therapy.

## 2. Materials and Methods

### 2.1. Planar Imaging and Automatic Segmentation

The proposed method is based on a single anteroposterior planar WBS performed after the administration of a radiopharmaceutical for therapeutic purposes. The geometric mean of the anterior and posterior views is computed and used for the automatic segmentation, according to the Magnander et al. [25] algorithm; this approach divides the body into two compartments characterized by high and low uptake. The method consists of identifying all those pixels whose counts overcome a given threshold counting parameter value, $C_{thr}$. For each threshold value, the number of uptake foci (NUF) is determined in the WBS, generating a distribution of NUF versus a threshold index (ThI) defined as:

(2) $$ThI=\frac{C_{max}-C_{thr}}{C_{max}}\;$$

where $C_{max}$ is the maximum pixel value in the WBS. The NUF is normalized (nNUF) to the maximum number of uptake foci and can then be described as a function of ThI ranging from 0 to 1. This distribution is then analysed to define a nNUF threshold value properly separating the two compartments.

### 2.2. Bone Marrow Dose Calculation

In the MIRD scheme, the dose to the BM is calculated as the sum of a self-irradiation dose, mainly including the energy release due to beta particles, and the dose due to photons coming from the low- and high-uptake regions, according with the relation:

(3) $$D_{\mathrm{BM}}={\tilde{A}}_{BM}S\left(r_{BM}\leftarrow r_{BM}\right)+{\tilde{A}}_{high}S\left(r_{BM}\leftarrow r_{high}\right)+{\tilde{A}}_{low}S\left(r_{BM}\leftarrow r_{low}\right)$$

The BM dose was calculated according to Equation (3), in which ${\tilde{A}}_{BM}$ can be obtained from ${\tilde{A}}_{low}$, as assumed in the Benua blood-based dosimetric scheme [7,30]. The dose imparted to the BM from skeletal lesions (SL) is mainly due to beta particles, whose contribution must be explicitly added according to:

(4) $$D_{\mathrm{BM}}={\tilde{A}}_{BM}S\left(r_{BM}\leftarrow r_{BM}\right)+{\tilde{A}}_{high}S\left(r_{BM}\leftarrow r_{high}\right)+{\tilde{A}}_{low}S\left(r_{BM}\leftarrow r_{low}\right)\;+{\tilde{A}}_{SL}S\left(r_{BM}\leftarrow r_{SL}\right)\;$$

in which ${\tilde{A}}_{SL}$ is the cumulated activity in the SL. The proposed calculation scheme, exploiting Equation (4), has the advantage of being based on the S-factor from the *OpenDose* project [29], which includes the specific dose rate for the average male and female computational phantoms, defined within International Commission on Radiological Protection (ICRP) publication 110 [31,32]. Both the detailed description of the anatomical characteristics of the phantoms and the possibility to concentrate the activity into one or more districts allow an accurate reconstruction of the dose imparted to target organs.

### 2.3. Estimated Pharmacokinetics

In the proposed method, the following calculation scheme has been applied: The functional form of the effective decay is guessed for the two compartments in which the patient’s body is automatically segmented and properly scaled according to a single measure for a shortening dose calculation. The total administered activity and the ratio between the activities in the low- and high-uptake compartments are considered.

For the high-uptake compartment, the time–activity curve is represented with a mono-exponential function:

(5) $$u_{high}=2^{-\frac{t}{t_{1/2}^{M}}}$$

with an effective half-life of $t_{1/2}^{M}$. The notation in Equation (5) corresponds to the Hänscheid approach [28], where u indicates the normalized time–activity curve. The time–activity curves for the low-uptake compartment are represented with a bi-exponential function in which the mean effective half-lives for the fast and slow components are indicated, respectively, by $t_{1/2}^{F}$ and $t_{1/2}^{S}$. The whole number of decays for normalized activity is then derived by integrating in time the following relation:

(6) $$u_{low}=\frac{1}{6}\cdot 2^{-\frac{t}{t_{1/2}^{S}}}+\frac{5}{6}\cdot 2^{-\frac{t}{t_{1/2}^{F}}}$$

properly scaled according to the WBS data. The relative weights of the fast and slow components were arbitrarily fixed according to experience; this is a parameter of the model, along with the effective decay half-lives. The activity assigned to the high-uptake compartment then reads:

(7) $$A_{high}\left(t\right)=S_{high}\times u_{high}=S_{high}\times 2^{-\frac{t}{t_{1/2}}}$$

where $S_{high}$ represents the reconstructed activity scale factor. Similarly, the activity retained in the low-uptake compartment is scaled by the factor $S_{low}$. A linear equation system for calculating the two scale factors, $S_{high}$ and $S_{low}$, is then given by:

(8) $$\left\{\begin{array}{c} S_{high}+S_{low}=A_{injected}\\ \frac{A_{high}\left(t=\bar{t}\right)}{A_{low}\;\left(t=\bar{t}\right)}=\frac{S_{high}\cdot 2^{-\frac{\bar{t}}{t_{1/2}^{M}}}}{S_{low}\cdot \left(\frac{1}{6}\cdot 2^{-\frac{\bar{t}}{t_{1/2}^{S}}}+\frac{5}{6}\cdot 2^{-\frac{\bar{t}}{t_{1/2}^{F}}}\right)} \end{array}\right.$$

The ratio of the activities retained within the high- and low-uptake compartments on the left-hand side of the second equation is given by the ratio of the corresponding counts per second (cps) measured by the γ-camera. A dose reference value can then be obtained without an accurate calibration of the machine or a manual contouring of the images.

### 2.4. Comparison with ^177^Lu-(DOTA-TATE) Literature Data

A first preliminary validation of the method was attempted by comparing calculated doses against literature data on patients undergoing PRRT. In particular, we compared our results on BM with those obtained with repeated planar images [23,24]. In [23], the self-dose is computed as:

(9) $$D\left(r_{BM}\leftarrow r_{BM}\right)={\tilde{C}}_{BM}\times \phi_{BM\to BM}\times \Delta \;$$

where ${\tilde{C}}_{BM}$ is the activity concentration in the bone marrow, which can be obtained from the corresponding low-uptake compartment activity concentration multiplied by a constant factor [23], $\phi_{BM\to BM}$ is the absorbed fraction for self-irradiation, approximately equal to one for the electrons, and Δ is the mean energy released for each decay, which was set to 147 keV in [24]. 

In the following section, we will show the results of a calculation for the doses imparted to the BM in a simulated scenario, with the given distributions for the activity retained by the low- and high-uptake compartments in the two computational phantoms (male/female). The mean effective half-lives for the fast and slow components of the low-uptake compartment were assumed to be equal to, respectively, 2.4 h and 61 h [23]. The mono-exponential process in the high-uptake compartment was parameterized with an effective half-life of 69 h [23].

### 2.5. Preliminary Validation on ^131^RAI

To carry out a first performance assessment of the proposed approach, a comparison of the dosimetric results obtained with the proposed method and the reference Benua approach [7] was investigated in five patients with DTC undergoing RAI therapy. Any discrepancy between the data obtained with the two approaches was further compared against the hematological findings.

The values of the dose imparted to the BM were calculated according to the approximated method described above. WBS measurements were performed with a dual-head γ-camera (Skylight; Philips) 7 days after RAI therapeutic treatment. The effective half-life for the high-uptake compartment was set to 16 h [33].

The values of dose imparted to the BM were then calculated according to the Benua method using the protocol described by Lassmann et al. [7], including at least five blood samples for the bi-exponential fitting of the plasma time–activity curve. Blood samples (about 3 mL) from five patients treated with RAI for DTC were taken at 2, 4, 8, 24, 48, and 168 h post-treatment and probed into a well counter calibrated for 131-iodine activity determination. The whole-body (WB) activity was then measured at discharge (usually 48 h post-administration) and one week after the treatment using an ionization chamber positioned one meter from the patient. Both the WB and blood data were employed, respectively, for exponential and bi-exponential regressions of the activity dismission curves.

According to the MIRD formalism of Equation (1), the dose to the blood can be calculated by summing the contributions of blood self-irradiation and irradiation from the rest of the body. Benua and co-workers [5,6,7] neglected the contribution from the high-penetrating gamma component in the blood self-term, assuming a complete absorption of the 187 keV electron, representing the mean-energy particle from the beta decay. This irradiation in one milliliter of blood corresponds to an absorbed dose per administered activity of $3\times 10^{-11}\;\mathrm{Gy}\cdot \frac{\mathrm{ml}}{\mathrm{Bq}\cdot s}=108\;\mathrm{Gy}\cdot \frac{\mathrm{ml}}{\mathrm{GBq}\cdot h}$, which agrees with detailed Monte Carlo simulations. On the other hand, the S value corresponding to the whole-body irradiation was approximated, including only the gamma contribution, as $S_{blood\leftarrow \gamma \;total\;body}\approx S_{total\;body\leftarrow \gamma total\;body}\;$ and was calculated to be $S_{blood\leftarrow \gamma \;total\;body}=0.00589\times \bar{g}/wt$, where wt is the patient’s weight in kilos and $\bar{g}$ is a geometrical factor depending on the weight and height of the patient. This S value may be replaced with a term not depending on $\bar{g}$, based on Monte Carlo simulation, obtaining the same results for almost every patient. The whole calculated mean absorbed dose to the blood with the Benua methods then reads:

(10) $${\bar{D}}_{blood}\left[\mathrm{Gy}\right]=108\times A_{0}\left[GBq\right]\tau_{blood}\left[h\right]+\frac{0.0188}{{\left(wt\right)}^{2/3}\left[kg\right]\;}\times A_{0}\left[GBq\right]\tau_{\mathrm{WB}}\left[h\right]$$

in which $A_{0}$ is the total administered activity and $\tau_{blood}$ and $\tau_{\mathrm{WB}}$ represent the residency times of the radiopharmaceutical, respectively, within one millilitre of blood and the whole body. Without specific uptake by cells, as is the case with radioiodine and bone marrow, the activity in tissues is comparable with the plasma activity content. The dose to the blood has been assumed to be a good estimate for the dose to the bone marrow for more than three decades in clinical practice. Hematological data were obtained in all patients at baseline and at one week and one month after treatment.

### 2.6. Inclusion Criteria and Treatment Monitoring

Patients were included in our survey based on their availability since there was no need to select an unbiased cohort of individuals treated with radioactive iodine. In fact, since this study is focused on the approximated dose calculation scheme that has been developed, there was no aim to build a clinical statistic, only to present calculations performed in practice. These can give a qualitative gauge of the accuracy of the method compared with traditional standards. This exploratory research can be the basis for large clinical studies assessing the relationship between a dose index calculated with the method presented here and the effectiveness and counterindications of a given radiopharmaceutical.

Patient characteristics and clinical data are summarized in Table 1. For each of them, hemograms including erythrocytes, leucocytes (granulocytes and lymphocytes), and platelets were performed at baseline and one week and one month after RAI treatment. Any decrease in the cell counts in all blood cell lines were noted. The availability of metastatic patients gave us the opportunity to provide a calculation example in the presence of skeletal lesions.

## 3. Results

### 3.1. Comparison with ^177^Lu-(DOTA-TATE) Literature Data

In Table 2, the dose to the bone marrow for a phantom without skeletal metastases was computed by the proposed approximation scheme for both the average male and female, considering different values for the initial administered activity and its percentage retained by, respectively, the high- and low-uptake compartments. Table 2 was obtained using the bone marrow self-irradiation of Equation (9) for comparison purposes with the method proposed by Svensson et al. [23].

As shown, for an administered activity of 7.4 GBq, the order of magnitude of the mean, upper, and lower dose values are comparable to those obtained in real patients with repeated WBS in the study presented by Svensson et al. [23]. In particular, the investigation by Svensson et al. [23] showed how the ratio of this percentage can be extremely patient-dependent, differing in some cases from the mean values (63–37%) even more than the extreme values presented here.

Table 2, reporting the results of phantom simulations, can be thought of as a calculation table to be eventually used in everyday clinical practice.

### 3.2. Preliminary Validation on ^131^RAI

The demographic and clinical characteristics of the included patients are shown in Table 1. Table 3 reports the mean blood/BM doses calculated with the Benua method and those obtained by the proposed approximated method in five DTC patients treated with RAI therapy. For simplicity, we assumed here that the S-factor accounting for the contribution of the high-uptake sector can be identified with the S factor describing the total-body irradiation by the body itself, as in the Benua calculations [7]. As shown, a good overall agreement between the Benua the approximated WBS approach can be observed. For three patients (patients #1, #2, and #3), the calculated doses to the blood are nearly identical. For patients #4 and #5, the WBS dose values differ by factors of three and five, respectively, compared with the Benua counterparts. The hematological data were then analyzed to understand if the values calculated with our approximation for patients #4 and #5 could be meaningful and to give a possible reason for the large magnitude of the biological effect observed at 1 month in patient #1.

**Figure 1:**
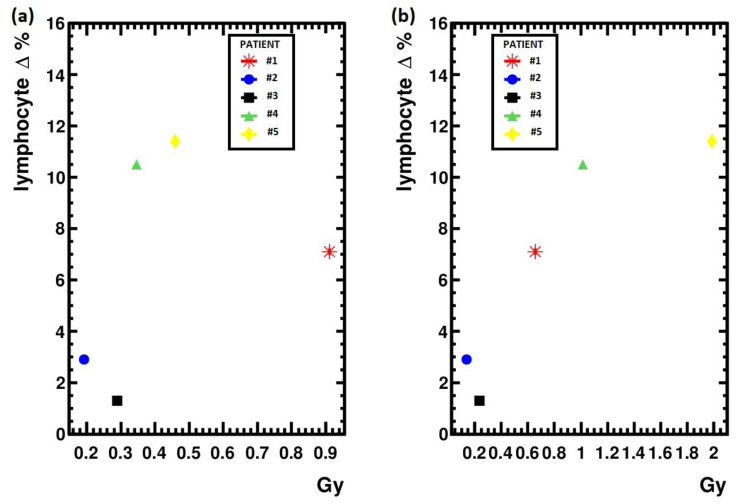
(**a**) Bone marrow doses calculated with the Benua method plotted against the lymphocyte percentage decrease at one week from treatment; (**b**) Bone marrow doses calculated with the approximated WBS method.

In Figure 1, the calculated doses to the blood are compared with the corresponding decrease in the lymphocyte percentage one week after the treatment. In fact, under the working hypothesis of an increased biological effect following a larger dose, the WBS approach provides approximated values whose ordering agrees with the observed hematological toxicity. 

Moreover, looking at the calculated BM values that differ from the blood counterpart in patient #1 due to skeletal metastases, the approximated doses seem to correlate better with the leukocytopenia observed in patient #1 and #5 and in patient #4 one month after the treatment. 

**Figure 2:**
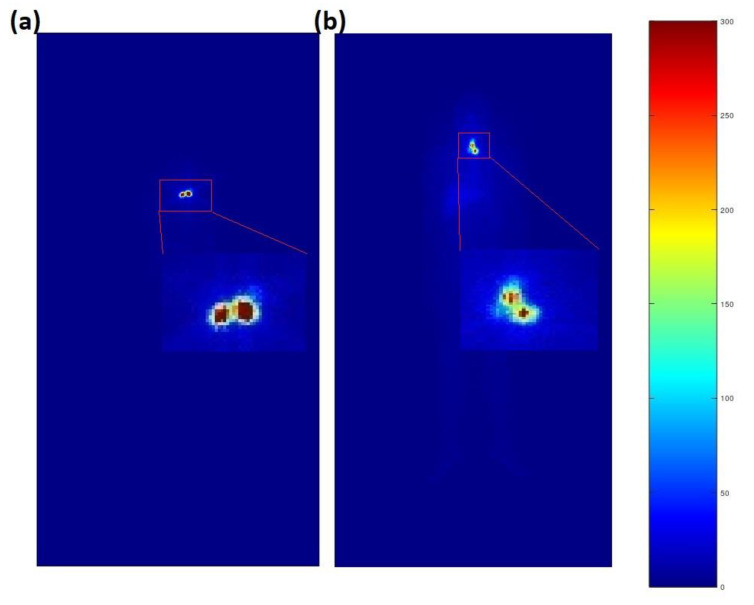
(**a**) Whole-body scan of patient #2; (**b**) Whole-body scan of patient #4. Colors represent the total count number for the corresponding pixel. The remnant/pathological high-uptake areas have been magnified.

The discrepancies between the WBS approximation and the classical dosimetric methods could be explained using the scintigraphic images reported in Figure 2, showing the whole-body scans of patients two and four seven days after receiving the therapeutic activities of 50 mCi and 100 mCi of RAI, respectively. In fact, the amount of activity within the high-uptake areas in patient four was not doubled with respect to patient two, determining a larger amount of activity retained in the low-uptake compartment of the body, as defined by the automatic segmentation algorithm. The difference in the calculated dose from the WBS could then seem reasonable.

## 4. Discussion

An approximated calculation scheme of the dose imparted to the BM from radionuclide therapy has been presented according to an automatic segmentation algorithm that was previously proposed [24,25]. The method is extremely fast and gives physicians the possibility to avoid contouring, allowing its massive application in everyday practice. Although it is based on a few approximations, the proposed approach could provide a reference framework for the calculation of the doses imparted to nontarget organs and lesions.

The method presented in this article borrows the single-time-point approximation suggested by Hänscheid et al. [28]. This approximation employs normalized activity curves in the form shown in Equations (5) and (6), which should not be interpreted as a detailed reconstruction of the variation of activity in time, comparable to that usually measured with repeated WBS or SPECT, but as an effective activity, $A_{eff}\left(t\right)=Su\left(t\right)$, whose integral approximates the real cumulated activity. For a mono-exponential time decay, using $A_{eff}\left(t\right)$ in place of its real counterpart, $A\left(t\right)=A_{0}e^{-\frac{t}{\tau }}$, induces an error, E, in the calculation of the total number of nuclear decays:

(11) $$E=\tilde{A}-{\tilde{A}}_{eff}=A_{0}\tau -S\tau_{eff}=A_{0}\left(\tau -\tau_{eff}w\left(\bar{t}\right)\right)$$

in which $w\left(\bar{t}\right)=A\left(\bar{t}\right)/A_{0}u\left(\bar{t}\right)\;$ is a weight function given by the ratio of the real and effective exponential at time-point $\bar{t}$, in which the WBS is acquired. Choosing $\bar{t}$ at about half the decay of the real curve, with the real time constant of the discharge process, $\frac{1}{2}\tau_{eff}<\tau$ < 2$\tau_{eff}$, one can restrain the error within 10% of the actual cumulated activity, $\tilde{A}$.

For a first qualitative and quantitative assessment of the method, preliminary results have been presented for two different therapies. The data in Table 2, which has been organized as a calculation table, refer to PRRT with ^177^Lu-DOTATATE. As discussed by Svensson and co-workers [23], patients treated with this radiopharmaceutical have a mean percentage distribution of the activity in the high-uptake compartments of 63% ± 11% (23–90%), with the remaining activity of 37 ± 11% (10–77%) found in the low-uptake compartment. It can be observed that the calculated dose values in Table 2 are comparable with the median dose to the BM of 0.19 Gy/7.4 GBq (range of 0.12–0.32) found by Hägmarker in [24] with repeated WBS for patients without skeletal lesions.

A quantitative comparison is illustrated in Table 3, presenting the blood/BM doses calculated with the Benua method in five patients with DTC undergoing RAI therapy and the analogous quantities obtained with the proposed approximated method. The hematological data were added to understand if the blood dose values calculated for patients #4 and #5 with the proposed approximation, differing from those obtained with the Benua method, could be meaningful. In fact, the Benua formula is assumed to provide a prudential estimate of the dose to the bone marrow, even if, in some clinical cases, its applicability has been controversial [34].

At the same time, RAI therapy has been shown to affect hematopoietic tissues, possibly inducing leukopenia and thrombocytopenia in 3–5 weeks [35] and a decrease in lymphocytes within the first two weeks after treatment [36,37]. The discrepancies between the WBS approximation and the classical dosimetric methods could be explained using the scintigraphic images reported in Figure 2, showing the WBS of patients #2 and #4, who received 50 mCi and 100 mCi of RAI. The pathological high uptake in patient #4 was not doubled with respect to patient #2, determining a larger amount of activity retained in the low-uptake compartment of the body, as measured with the automatic segmentation algorithm. 

Moreover, as reported in Figure 1, the doses imparted to the blood calculated with the approximated method correlate better with the decrease in the patient leukocyte percentage number observed one week after the treatment, which is then reasonably linked with the damage to the circulating cells. Finally, even the correlation of the dose imparted to the BM, resulting from adding the contribution of eventual skeletal lesions to the dose imparted to the blood, appears more meaningful for the approximated scheme discussed in this paper, as can be seen by comparing the hematological findings at one month after the treatment, as reported in Table 3 with corresponding dose values.

A shortcut of the method exploiting Equation (8) in the dosimetry of orally administered radiopharmaceuticals, such as RAI therapy for DTC, lays in the lack of quantification of the activity loss by gut clearance. In this case, neglecting the rising part of the uptake curve using a mono-exponential fit can lead to a significant overestimate of $\tilde{A}$, increasing with the administered activity. This effect can explain the higher dose values found for DTC patients treated with higher RAI activities. Although the self-calibration apparently enhances the sensitivity of the single WBS method to the activity content in the low-uptake compartment and its correlation with the blood and BM effects, in RAI and orally administered therapies calculated dose values that might be considered as a reference dose index. An accuracy such as that reached with ^177^Lu-(DOTA-TATE) could, however, be obtained by changing the functional form of the normalized activities in Equations (5) and (6), using the difference between two exponentials to simulate the slow uptake of the radionuclide. In perspective, this improvement in the method will be investigated and tested against a larger set of clinical data.

## 5. Conclusions

An approximated calculation scheme of the dose imparted to the BM from radionuclide therapy has been presented using the two-compartment approach from a single post-therapeutic (planar) WBS. 

The simple calculation scheme could be introduced into everyday clinical practice. Its routine use would allow the comparison of data coming from larger cohorts of patients, even from different centers, eventually enabling the establishment of their correlation with some of the acute and long-term effects of radiation in nuclear medicine therapy. However, on the path to such a wider exploitation, there is the need to perform a significant experimental assessment, analyzing data from many patients.

In perspective, this simplified approach could be adapted to the evaluation of the diagnostic (dose) reference level [38] in hybrid-PET examinations, whose exploitation in preclinical patients and the follow-up of oncological patients has dramatically grown in the decade since their introduction in clinical practice [39].

## Figures and Tables

**Table 1:** Demographic and clinical characteristics of patients.

Patient	Age (Years)	Sex	Administered Activity (mCi)	Histological Type of DTC	Tg Level on RAI (ng/mL)	Stage of Disease (I–IV)
1	77	M	122	Follicular variant of papillary type	8361.62	IV
2	41	M	50	Papillary type	2.15	I
3	47	F	50	Follicular type	0.23	I
4	40	M	100	Papillary type	0.36	I
5	61	M	150	Papillary type with sclerosing aspects	3.08	IV

**Table 2:** Bone marrow doses calculated for different administered activities versus the measured activity ratios between the low- and high-uptake compartments for ^177^Lu-DOTA-TATE using the bone marrow self-irradiation term of Equation (9), as done in [24].

Administered Activity (High- and Low-Uptake Compartment Activity Ratios)	5.6 GBq/150 mCi	6.5 GBq/175 mCi	7.4 GBq/200 mCi	8.3 GBq/225 mCi
57–43%	0.158/0.185 (M/F)	0.183/0.215 (M/F)	0.208/0.244 (M/F)	0.234/0.274 (M/F)
60–40%	0.150/0.176 (M/F)	0.174/0.204 (M/F)	0.198/0.232 (M/F)	0.222/0.261 (M/F)
63–37%	0.142/0.167 (M/F)	0.165/0.193 (M/F)	0.188/0.220 (M/F)	0.211/0.247 (M/F)
66–34%	0.134/0.158 (M/F)	0.156/0.183 (M/F)	0.178/0.208 (M/F)	0.199/0.233 (M/F)
69–31%	0.127/0.148 (M/F)	0.147/0.172 (M/F)	0.167/0.196 (M/F)	0.188/0.220 (M/F)
72–28%	0.119/0.139 (M/F)	0.138/0.162 (M/F)	0.157/0.184 (M/F)	0.176/0.206 (M/F)

Values are reported in Gy.

**Table 3:** Dose imparted to the bone marrow of five patients treated with 131-iodine, calculated with the Benua method (Equation (10)) and the approximated approach, based on planar WBS, discussed in this article. Patients’ selected biochemical data are compared with doses.

Patient	Age (Years)	Sex	Administered Activity (mCi)	Benua Blood Dose	WBS Approach Blood Dose	WBS Approach Skeletal Metastases Dose	Lymphocytes (% Decrease at One Week)	Thrombocytes (% Decrease at One Month)	Leukocytes (% Decrease at One Month)
1	77	M	122	0.911	0.656	1.918	7.1	49.4	2.7
2	41	M	50	0.192	0.139		2.9	17.7	1.9
3	47	F	50	0.289	0.236		1.3	8.3	0.4
4	40	M	100	0.346	1.014		10.5	19.9	32.0
5	61	M	150	0.459	1.987		11.4	35.2	0.5

Dose values are reported in Gy.

## Data Availability

The data presented in this study are available on request from the corresponding author. The data are not publicly available due to privacy.
